# Personal Experiences vs. “Scientific Proof,” as Factors Bearing upon the Amalgam Question

**Published:** 1898-05

**Authors:** 


### COMMUNICATIONS.

### Personal Experiences vs. "Scientific Proof," as Factors Bearing Upon the Amalgam Question.

That there are many subjects of interest to dentists which have been worn well-nigh threadbare from repeated discussion, the literature of our profession furnishes abundant evidence, and were it not for the objections which are being urged by so many physicians against the use of amalgam, and which we realize are commanding public attention in almost every city and town throughout the land, there would seem to be no reason for attempting to add anything further to what has been already said or written upon the subject of amalgam as a filling material.

It is only within a few years, comparatively, that the objections to amalgam, from a medical standpoint, have commanded much attention from the dental profession. The impression has prevailed among dentists, and apparently still exists everywhere, that a certain class of physicians are attempting, in a measure, to dictate what the dental profession shall or shall not use as suitable filling materials. The result has been to stir up a prejudice and bitter opposition against such physicians; an ill-concealed effort on the part of dental societies, through their active members or officers, to prevent any debate upon these objections to amalgam in open meeting, and a more determined purpose than ever on the part of a large majority of dentists that neither any portion of the medical profession nor an intelligent public opinion shall force them to discard the use of amalgam until they shall become agreed, by unanimous consent, that it is for their best interests to do so.

Having been one who never hesitated to use amalgam whenever it seemed best to do so, having entertained the same opinions of physicians who objected to amalgam that have been expressed by others in dental meetings, and with no particular desire to satisfy himself whether these objections to Amalgam were well founded or not, until practically compelled to do so, the writer can understand perfectly the feelings of a dentist when confronted by a patient who says it is the wish of his physician that all amalgam fillings be removed, many of which the dentist himself has very likely inserted, and which are apparently doing good service.

There can be but one outcome to any controversy with physicians in which a purpose not to yield to certain instructions to their patients is manifested by members of our profession. We shall be forced either to yield to their instructions upon certain questions at issue, or to see the patients go elsewhere.

To give expression to the belief that the arguments advanced by physicians will always admit of intelligent consideration among dentists and to give a portion of the abundant testimony received from physicians and patients relative to the ofttimes harmful effects of amalgam fillings is the purpose of the present article.

If the seemingly "popular" prejudice which has been already awakened in the public mind against these feelings shall result in the finding of some *new* material which shall be the "ideal" substance we have all been hoping would some day be discovered, we can at least rejoice in the blessings which such a discovery would prove to humanity.

Of the good and bad qualities of amalgam, as a filling material, much has already been said. Observation and experience have convinced the writer it is the *poorest possible* material. The inability to successfully manipulate gold in a large percentage of cases where gold could be used, and for which the patient would be willing to pay, furnishes the incompetent and lazy operator a convenient excuse for arguing that in no other better or more suitable way can a great number of teeth be saved than by the use of amalgam.

The argument is further advanced that many people can neither afford to have their teeth filled with gold nor the repeated expense of having cement or gutta-percha fillings frequently renewed. The latter argument is a plausible one, and it took the writer some time to realize how often it could be controverted. There is no disputing the fact that amalgam will remain in a tooth a much longer time than either gutta-percha or cement, but the appearance of the great majority of amalgam fillings coming under our observation from day to day, along the margins of which an instrument varying in size from an exploring needle to a good-sized excavator can be easily inserted, is in itself sufficient to raise the question *how* well these fillings are saving the teeth, and an examination of the cavity upon removal of such fillings is generally a sufficient answer to the question. We have only to compare cavities from which cement or gutta-percha fillings have been removed and which simply need renewing, to get pretty accurate knowledge as to the relative merits of this class of fillings over amalgam, and it is but fair to take, by way of comparison, cavities in both cases that have been prepared and filled by equally painstaking and conscientious operators.

As an expression of the views which I find are quite common among people who are convinced from personal experience that amalgam does *not* save teeth as well as does gold, cement or gutta-percha, and who have no particular opinion to offer concerning the special objections offered to it by physicians, the following letter from one of Boston's successful business men may be of interest:

BOSTON, October 21, 1897.

DR. CHARLES H. TAFT, 16 Arlington St.

DEAR SIR: You ask for my opinion about the use of amalgam for filling. As to its merits medically, I have none to offer. That is something you will have to fight out with the doctors. From a layman's standpoint, however, as to its merits physically I have a very decided opinion, so far as my own experience goes. In figuring the matter up within a year or two it became evident that I must have from 125 to 150 teeth, or that some of them were being re-filled over and over again. Upon consulting memory and the record I found that this latter was unquestionably the case with those in which amalgam had been used, and that in most, if not all, of these teeth the process was still being repeated at irregular intervals. Whatever may have been the case in boyhood, for the last twenty or twenty-five years I have employed only those dentists of acknowledged reputation. It may be also that in boyhood the question of so-called economy may have been suggested on the part of the dentist, and without the writer's being informed as to how poor an economy it really was. Of late years the responsibility has been solely on the part of the operator, and never, until I came to your hands a year or two ago, has a dentist even suggested that there was any point to be considered except that of appearances and, perhaps, the first cost, and as a result I have been allowed, year after year, for two or three decades to have half my teeth at least filled with a material which I am convinced, from my own experience, was worse than no filling at all. Apparently in every case the filling has shrunken, the decay begun again unnoticed and necessitating finally a removal of the filling, an enlarged cavity, and a plugging up with the same material, with the same process to be gone through with again, with the consequent expense, pain, loss of time, and the tendency toward the final irreparable loss of the teeth themselves, if this condition had not been noticed before it was too late.

Now, perhaps I may not be fully informed in the matter, but viewed from the present standpoint, those who use that material for filling are, to speak plainly, either fools or knaves. Fools, if after going through the regular course of instruction which any modern medical school ought to provide, and having observed what must have been the experience of thousands of people, they are unable to reason out so simple a relation between cause and effect. Knaves, if knowing the above, they allow their patients, either with their suggestion or without it, to go on using this form of filling. For no man fit to be out of a lunatic asylum would ever, voluntarily, allow such stuff to be put into his teeth if the simple facts, as might be expressed in a single sentence, were made known to him. I regard it an outrage that those in a so-called learned profession will thus impose on the public, who must be misinformed as to the technicalities of the art. If we, or any other mercantile establishment of any name or nature, carried on our business with the public along such lines we would land in bankruptcy as soon as the fact was half appreciated, and we would well deserve the obloquy of the whole human race. I find, upon inquiry, that I am not the only one who has begun to wake up to the fraud that has been practiced upon him, and if my experience is at all common, as I suspect is the case, and the public once get their eyes open to it, you will find there will be a howl all along the line, and it will require more sophistry than I have yet heard to meet the explanation that will be demanded. When that circus begins I hope I shall be around to take a hand in the fracas.—From a Long-suffering, but now Belligerent Victim.

But leaving out of the discussion all that might be said by those of us who differ as to the merits of amalgam as a filling material, there still remains to claim our attention the objection which physicians have to offer, to-wit: that, because of the mercury which these fillings contain, they are found to be continually acting either as a direct poison to the patient or as an obstruction to the action of well-indicated medicines in the cure of the sick. This objection, if well-founded, is a most important one, and can not be ignored by members of our profession.

Experience has taught many physicians that it is often useless to attempt to cure a patient with a chronic disease until all amalgam fillings have been removed. Experience has taught the writer that it is useless to argue with a patient who is determined to follow the advice of the physician against the foolishness of having such work done. Experience has also proved to the writer that in a large percentage of cases the removal of amalgams has been followed by results eminently satisfactory to physician and patient. It has proved that medicines have a much quicker and more permanent curative effect, and that patients go about among their friends telling the latter to what the cure of their troubles was due.

Whenever interesting cases are reported at dental meetings where marked improvement in or cure of some chronic trouble has followed removal of amalgam fillings, the question is sure to be asked, "Well, how do you *know* that the cure was due to removal of these fillings? You can not prove that there is any free mercury in the system, and until you can demonstrate this fact in some scientific manner I will not believe that the amalgam fillings had anything to do with it."

It is, of course, impossible to present within the limits of a magazine article an exhaustive argument showing why amalgam fillings may often prove a source of evil. The most that can be done is to point the way to an intelligent method of reasoning to all who are willing to lay aside prejudice and to meet the objections to amalgam from a medical standpoint upon their merits, and to give some of the abundant testimony which the writer has received from both physicians and patients, and which speaks for itself.

To furnish any "scientific proof," in the ordinary sense of the phrase of the injurious effects of Amalgam fillings, is quite impossible; nor do we, as dentists, really need any such proof. There are many things which can be readily demonstrated, but which would not actually *prove* anything. As the Rev. Phillip S. Moxom, D.D., said in a very able argument for immortality, presented before the World's Parliament of Religions at the Columbian Exposition of 1893, in which he took the ground that any proof of immortality, according to modern scientific methods, was impossible. "Even the miracle of a physical resurrection, while it would be a demonstration of revival from death, would not prove immortality, for it would be a transaction quite as much on the plane of the material as revival from a swoon, and as death supervened once, it might supervene again. Demonstration of immortality lies solely in the sphere of personal experience. The man who from blindness attains sight has demonstration of the reality of vision, but even he could not demonstrate that reality to mortal men. So only the soul that has entered upon immortality has demonstration of that supreme reality; and though one should rise from the dead, yet would he be incapable of demonstrating immortality to mortal men."

And this is the situation in a nutshell with regard to the injurious effects of amalgam fillings. Any demonstration of the fact that amalgams are ever a source of evil *must* lie solely within the sphere of *personal* experience.

Could we discover the presence of free mercury in the system by the aid of scalpel, microscope or otherwise, it might demonstrate an interesting *fact*, but its mere presence there would not prove it to be a source of harm to the patient. It would be difficult to prove in the same manner the presence of "free ivy" in the system of one who had but passed near a bed of the poisonous plant, and yet who developed unmistakable symptoms of ivy poisoning. We can demonstrate, for example, the presence in certain throats of the Klebs-Loeffler bacillus, but such demonstration does not prove that diphtheria is either present or will invariably follow because of the presence of these micro-organisms. Their presence has been often noticed in the throats of perfectly healthy people, and the reason of their not being a source of harm is because there is no impaired condition of the system or the vital force which makes it susceptible to any harmful influence of these organisms. The same may be said of tuberculosis, and of all other diseases which are popularly supposed to be *caused* by these germs. The mere presence of the germ of typhoid can not produce typhoid. The system must be in a condition to make the germ operative for harm. The mere presence of mercury, whether combined with one or more metals, or if found free in the system, could not produce characteristic symptoms of mercury poisoning unless the system, for some reason or other, was "susceptible" to it. The presence of pus about the socket of a wisdom tooth does not prove the existence of a dead pulp, for it may be due to causes attending a difficult eruption of the tooth.

The mere presence in the stomach of some common article of food, such as the strawberry, cannot be said to prove *why* some people cannot indulge in the fruit without almost immediately showing evidence of acute distress. Demonstration of the presence of quinine in the system does not prove, except as it lies within the sphere of personal experience, that the drug can cause or cure chills and fever when taken in either large or curative doses. The presence of pulp stones does not prove them to be the cause of facial neuralgias or other troubles of reflex nature simply because we happen to find that such troubles often disappear upon removal of the offending cause; for it frequently happens that neither removal of the pulp stone, nor the tooth itself will effect the desired result. And yet we never think of asking the man who reports a cure of facial neuralgia upon the finding of a pulp stone and its removal, or that of the tooth, to furnish us with some really "scientific" proof that such was the cause before we are ready to believe it.

It may be said, then, that we cannot actually prove to others the things which have been cited as illustrations according to what we commonly call *scientific* methods. It is fair to presume, therefore, that no one who gives the matter serious thought will say that we can ever *have* accurate proof of the fact that drugs have either toxic or medicinal proprieties, save as such properties are demonstrated in the sphere of *conscious, personal* experience. No effort that the writer is aware of has ever been made by any member of our profession to prove by "scientific methods" that amalgam fillings are *not* very often a source of harm. Statements to the effect that dentists have been filling teeth with amalgam for sixty years or more with no serious results, so far as is known, may be abundantly conclusive to a great many minds, and as proving the fallacy of the arguments advanced by our friends of the medical profession; but until these arguments can be disproved by that kind of scientific proof for which the profession is so earnestly clamoring, it may be well to listen to the testimony of those who have answered the question, Are amalgam fillings ever injurious? judging from "personal experience."

But to go back to the question of free mercury in the system. Suppose for a moment we could prove its presence as coming from amalgam fillings; its presence would not explain to us the process which goes on when functional disturbance takes place as a result of its presence, and manifesting itself in symptoms which are plainly characteristic of mercury. No more would "free" ivy (supposing we could prove its presence in the system) explain either the invisible force emanating from the plant and poisoning those who are susceptible to it, or that process, as we have just said, which goes on when functional disturbance takes place as a result of its presence.

In any discussion of this subject the question of the varying susceptibility of people to different drugs or poisonous influences must play an important part.

The fact that a single person believed that amalgam fillings were often a source of evil would not be any real evidence that such is the case, nor would it be any distinct *proof* if we found this idea becoming universal. When we do, however, find an idea becoming quite generally entertained by a large number of intelligent people in any community, gathering strength day by day, and when that idea is either the expression of an instinct or a deep-seated conviction, born of experience, observation, or a logical train of reasoning, then the very universality of such an idea becomes of great evidential value. If we have visible demonstration of a great many people becoming cured of chronic troubles, sooner or later after the removal of amalgam fillings, we reasonably infer that the amalgams had been acting as an *obstruction*, at least to the action of medicines, and that fact, also, would have evidential value merely—of scientific *proof* that such was the case there would be none.

That free mercury is expressed into the tubuli of the dentine when we insert an amalgam filling without taking the precaution to coat the cavity with some sort of varnish has been demonstrated by our friend Dr. R. R. Andrews, of Cambridge, Mass., whether or not the system can be affected by the inherent action of the mercury through such channel, opens up an interesting question and one that will bear intelligent investigation.

In this connection, too, it would be interesting if some one would discover and explain the "how" and the "why" drugs ever exert either toxic or medicinal action. It is not enough to say that because a person develops symptoms of arsenical poisoning that there is "free" arsenic in the system. A post-mortem, it is true, may reveal it in a person who has taken an over-dose of the drug. But will an examination, post-mortem or otherwise, reveal, "free" arsenic in the system of a person who is poisoned by sleeping in a room the wall-paper of which contains arsenic?

It is enough to say that because arsenic is known to be a valuable remedy in curing certain kinds of cold or influenza we have only to put a certain amount of it into the system to accomplish this result.

In neither case is the *action* or *force* of the drug explained, either in disturbing or restoring the equilibrium of the vital force, for this is what the physician has really to do with in the practice of his profession. We measure the ability or skill of a physician in his chosen specialty by the manner in which he forces a disturbed condition of the vital force to regain its equilibrium by the action of well-indicated medicines. If the vital force quickly responds to the action of medicines in the manner that he who practices the art of healing intelligently knows it should respond we have every reason to believe that it is due to the medicine, and *not* to what some people flippantly call the "imagination" of the patient.

Let us, therefore, accept *results* and *facts* as we find them. If it is found that people actually do get better or are cured of chronic troubles after their amalgam fillings have been removed, let us quit getting excited and calling physicians cranks, and the patient a person who has been hypnotized into believing these fillings were doing him harm, and learn all we can about drugs (including mercury) and their action. It is infinitely more profitable and interesting to study a subject like this, than it is to cover our ignorance behind a mass of epithets hurled at members of a kindred profession, at the same time offering nothing worthy of an argument against the objections which are becoming so widely entertained among the laity throughout the land to the use of amalgam.

There are *many* things we have yet to learn, and a little investigation upon almost any subject will often reveal things of interest which spur us on to still further research. The remarkable penetrating power of crude mercury may never have occurred to many of us, but if we split open one of the ordinary wooden bottles found upon almost any operating cabinet which has contained mercury we shall, with the aid of a magnifying glass, see little rivulets of mercury running all through the fiber of the wood. This is mentioned as an interesting fact which any one can observe for himself, rather than because of any special bearing it has upon the subject we are considering.

In conclusion, the writer has found, from his own experience, that an excellent method of preparing himself to discuss the injurious effects of amalgam fillings intelligently with both physicians and patients is

1. To study the action, not only of mercury, but of other drugs as well, both as to their toxic and their medicinal properties.

2. To ascertain whether or not there is a law or definite principle governing the action of drugs, and if there be such a law or principle, to make careful study of its operation.

3. To take a drug and test it upon one's self, or others, with a view to becoming satisfied whether or not there is what has been called a *dynamic* force or energy inherent within it and possessing actual curative properties.

4. To study the vital force, its nature or essence, and to find out whether it is this force or the material organs and tissues of the body themselves upon which the dynamic action of a drug, rather than the crude drug itself, is to be directed.

5. To study how this vital force may be thrown out of normal equilibrium—in other words, its susceptibility to drugs or other harmful influences.

The writer of this paper makes no claim of having scientifically *proved*, in the common acceptation of the phrase, that amalgam fillings are often a source of evil (and probably much oftener than most of us are aware of), or that the question is one which admits of such proof. He merely makes the claim of having abundantly proved, to his own satisfaction, during the past seven or eight years, the fact that they very often are a source of harm—such proof resting to a considerable extent, of course, upon what we have seen is necessary in any consideration of the question, to-wit: the *conscious, personal experience* of others.

Dental literature offers nothing that can be called a record of cases—to which reference can be made at any time by those who are interested to make special study of the subject—where any evidence is recorded of the injurious effects of amalgam fillings.

The importance of having ready access to any evidence from patients bearing upon the subject is easily seen, and the following letters are therefore appended as a portion of the abundant testimony in the writer's possession which he has begun to accumulate from physicians and patients in the hope that their perusal will be of interest to all readers of THE DENTAL REGISTER. No permission to publish these letters having been either requested or granted, the identity of the writers is withheld where it has been felt not only right but proper to do so.

CHARLES H. TAFT, A.B., D.M.D.

16 Arlington St., Boston, Mass.

---

CHICAGO, ILL., October 3, 1897.

DEAR DR. TAFT: Your favor of the 1st inst. requesting my views upon the effects of amalgam fillings has been received. Presuming that personal statements are of small value, unless based upon demonstrable facts, I shall beg your indulgence and narrate a few cases from my private practice:

Mrs. C., a woman of sturdy Massachusetts parentage, of exceptionally fine personal and family history, consulted me for a general physical breakdown. She was troubled with insomnia, nervousness, loss of appetite, and an almost intolerable neuralgia of the face and head. Her vision was considerably disturbed. The case having resisted the treatment of some noted specialists, I gave the case a most cautious prognosis. An examination revealed the presence of six or more amalgam fillings. These were removed, not without serious difficulty. A prompt and perfect cure followed. It is proper to say that some constitutional remedies were employed, but I do not credit them with more than a secondary influence in bringing about the cure. Before the removal of the mercurial fillings no medicine had given the slightest material benefit.

This case is fairly representative of a score of others all coming within my personal knowledge, with the details of which I

need not weary you by repeating at length. I have known exhausting facial neuralgias, sympathetic errors of vision, chronic ulceration of the mucous membrane of the mouth and various incurable symptoms of a constitutional character to disappear as if by magic upon the removal of the exciting cause, namely, one or more amalgam fillings. I recall one remarkable case, that of a young woman residing in a distant State, in which I made a brilliant cure by ordering the removal of the mercurial fillings from her teeth. It is not too much to say that this patient had been reduced to a state of chronic invalidism by these fillings. I had never seen her, but the symptoms she gave were so clearly indicative of mercurial poisoning that I had no doubt of my ground when I declared my belief as to the cause of her troubles. Her family regarded the cure as a very remarkable evidence of my medical skill, whereas it was merely an application of the rules of sound hygiene. Medicine, unfortunately, got most of the credit. Superstitious faith in bottled wisdom is not yet extinct, even amongst members of the medical profession.

In Dennis' *System of Surgery*, Vol. 4, p. 20, you will find the record of a case of epithelioma of the mouth, with the inevitable conclusion that the cause of the tumor was an irritation due to the wearing of a red rubber plate. I can testify, from personal knowledge, to the deleterious influence of this material. It is admitted that neither gold nor aluminum causes irritation when worn in the form of a plate.

I do not believe that all amalgam fillings produce mischief; in fact only a small percent of these fillings do produce serious trouble, calling for special treatment; but that mercurial fillings do cause pronounced pathological conditions a great many times there is not the slightest reason in any candid mind to doubt. A case of Bell's paralysis occurs to me. A young man of twenty-two, of excellent habits, a non-user of tobacco or stimulants, applied to me for relief from one of the most clear-cut cases of this trouble that I have ever seen. Weeks of careful treatment resulted in no benefit. Suspecting the presence of amalgam, I found a single large filling in an upper molar. It was removed and replaced with gold. The cure was prompt, without a single dose of medicine to confuse the issue. Unfortunately, I have kept no available record of these cases of amalgam poisoning, but I am confident that I have cured more than twenty cases of violent neuralgia of the jaws and face by removing the amalgam. The dental profession is not altogether at fault in not recognizing more widely the mischief wrought by mercurial fillings and plates. One reason for this apathy is found in the fact that these troublesome cases report to the physician rather than to the dentist. In many instances these fillings are carried for years before trouble appears, and often the connection between cause and effect is lost.

In conclusion, I beg to express to you my thanks for your persistent and effective labors in bringing this matter before your profession, and hope most earnestly that sufficient interest will be aroused to insure a most careful study of the abundant and conclusive evidence now in hand. Dental surgery has become one of the first of useful modern arts, and I am confident that the final verdict of the profession will declare for the gold standard in dentistry, as well as in finance.

Yours fraternally,

HOWARD CRUTCHER.

---

BELLASYLVA, PA., October 11, 1897.

CHARLES H. TAFT, D.M.D., Boston.

DEAR DOCTOR: Your favor of the 6th inst. was forwarded. I return to Brooklyn end of this week. The question before your Society, "Are Amalgam Fillings Injurious?" I can answer affirmatively from an experience in my own family. My niece suffered from the so-called silver (quicksilver) fillings not only with disordered digestion and its consequences for a long time, but also from a well-marked, offensive odor which, to say the least, was very unpleasant not only to herself, but also to those approaching her. As soon as the fillings were removed the odor disappeared and she began to get better, generally. The symptoms were plainly those of mercury, as known to the homoeopathician by Hahnemann's provings. The refilling with gold took, of course, a long time, and, besides being quite painful, cost a little money, but she improved from the time that all these fillings were removed. I have also noticed in others of my patients the evil effects of amalgam fillings, and invariably advise their being replaced with gold. It is very desirable indeed that an invention should be made which dispenses with all metallic fillings, because even gold is liable to affect sensitive persons and I suppose all metals would do the same, namely, satisfy their pathopœtic tendency in sensitive organisms. I hope, in the course of time, a cement will be invented, which, being inert, will cling to the bony substance of the tooth, uniting with it indissolubly. In this anticipation, I remain

Yours fraternally,

B. Fincke, M.D.

---

New York, 62 W. Forty-ninth St., October 11, 1898.

Charles H. Taft, D.M.D., 16 Arlington St., Boston, Mass.

Dear Doctor: Your letter received, asking me whether I am prepared, from observation of cases, to answer the question, "Are amalgam fillings injurious?" My answer is, Yes. Numerous cases in illustration have come under my care. Patients exhibiting symptoms of mercury used to puzzle me when they would not yield to treatment. They had sore mouths, sometimes too abundant saliva, and occasionally constitutional symptoms. At length my attention would be called to the teeth—perhaps not on account of pain or discomfort in them, but by some accidental occurrence—and they revealed cavities filled with amalgam. It was some time before I came to insist upon these fillings being removed. Now I do in all cases, abundant experience having convinced me of the necessity of it. I found it impracticable to control the symptoms otherwise. They might, in some instances, be relieved, but nevertheless came back. Many would persist with very little amelioration from treatment until the fillings were removed, and not before.

It was interesting to watch the mental processes of some of my patients in the meantime. As a rule, people do not like to be told that the fillings in their teeth are faulty and mischievous, and must come out. The question of expense counts with many, also time and discomfort, and then the disturbed and calculating dentist has his say and influence. The one who put in those fillings is actively opposed to the physician who criticises his work. When "pshaws" and ridicule and opposing statement are of no avail, he knows how to fall back upon the declaration that it is impossible to properly fill those large, bad cavities with anyting but amalgam. That is a pretty stiff argument against me—for a short time. It causes the patient to halt. My rejoinder is, that if a tooth was to be considered only by itself, it is cheerfully granted that amalgam is not only permanently effectual but innocent; but that the tooth is a part of a complex organization, which is alive and sensitive to different agents, markedly so to mercury; that I am certain of its harmfulness (the mercury in the amalgam), and that the dentist in question having confessed his inability to do the best work, I must now be allowed to recommend a true artist who can, and will, repair teeth with material, and in a manner to suit me. Then there is a different attitude. Members of the dental profession now seldom recommend amalgam fillings to any of my patients. When brought to account for doing such work they say, "Oh! I didn't know you were a patient of Dr. Carleton. Of course, you can have something else."

Yours truly,

EDMUND CARLETON.

---

BOSTON, 138 Marlboro St., October 11, 1897.

DR. CHARLES H. TAFT, Boston, Mass.

MY DEAR DOCTOR: Yours of October 7th duly received. The question to be discussed at the dental society is one of great interest to me, and I should be glad to go to a great deal of trouble, if I could help to hasten the time when dentists would refuse to use amalgam fillings. I have a few cases that would, I believe, convince the most sceptical; but, as a rule, I have found that diseases present before the amalgam was put into the teeth were aggravated, and that they would not yield to the indicated remedies until the amalgam was removed. I have found this especially true in chronic catarrhs, so that for the last three years I have refused to treat such cases, unless the patients would have the fillings removed. In regard to the question why these fillings prove harmful in some cases and not in others, I know of no satisfactory answer. We know that certain persons cannot drive past a spot where poison ivy is growing without being poisoned, while another can handle the ivy itself with impunity. One person will have convulsions if he only inhale for one instant the fumes of camphor, while another can inhale it daily for years without any apparent results following. We cover our ignorance by calling this idiocy—crazy or susceptibility; but that is no explanation. The fact, however, cannot be denied. So a person very susceptible to mercury will get results from amalgam fillings at once. Those less susceptible will feel it only after several months or years, while others, apparently, are not at all susceptible, and no evil results follow its use. I enclose the reports of two cases. I have written them out rather more fully, perhaps, than necessary. The most striking case that I have ever had was reported by Dr. Kimball at one of the meetings of the dental society several years ago. If you would like any further information in regard to either of these cases I should be very happy to give it to you.

Yours very sincerely,

FRANCIS M. MORRIS.

CASE I. In 1873, Mr. L—, a pastor of one of our city churches, consulted me in regard to his throat. He had been troubled for several years with hoarseness after preaching a sermon, and sometimes he would lose his voice entirely for several hours. He also had frequent attacks of follicular tonsillitis every winter, and he feared that he might be compelled to give up preaching if he did not get relief. He had had treatment from both schools of medicine with only temporary relief. As I examined his throat I noticed that he had a number of large amalgam fillings in his teeth. He thought several of them had been in several years before he noticed any trouble with his voice. I told him at once that I would not attempt to treat him unless he would have the fillings removed. He thought he could not do this and I saw nothing more of him for several months, when he came into my office with his wife. He told me that he went to Dr. — a few weeks after he had consulted me, who also refused to treat him until the fillings were removed. He therefore yielded and put himself into the dentist's hands and had every amalgam filling removed. In 1896 I saw the gentleman again. He told me that he did not suffer with his throat at all, and upon examining it I found that the enlarged follicles had entirely disappeared from the pharynx, and that the mucous membrane appeared to be in a perfectly healthy condition.

CASE II. Miss C— consulted me in regard to a very troublesome sore on her tongue which she feared was a cancer. Eighteen months before she came to me she had had an amalgam filling put into her second right inferior molar. The filling was rough and scratched her tongue. After a few days she returned to the dentist who made the filling smooth, but the tongue did not heal entirely, and soon she noticed a ragged looking spot not much larger than a pin head. This gradually increased in size and became very painful. She was sure it was cancer but would not consult her physician, because she did not want her fears confirmed. When she came to me I thought, at first sight, that she had made a correct diagnosis of her own case, for the middle third of the right side of the tongue seemed to be broken down into a malignant looking ulcer. I sent her at once to a dentist and had the filling removed. She remained under my care for about a week when the pain had entirely left her tongue, the ulcer looked much less malignant and she returned to her home in New York. I made no local application. She had no further treatment, and after a few months wrote me that her tongue was entirely healed.

---

FREDERICK O. PEASE, M.D.,
Professor Materia-Medica and Clinical Medicine,
Dunham Medical College.

CHICAGO, October 9, 1897.

DR. CHAS. H. TAFT, Boston, Mass.

MY DEAR DOCTOR: Your favor of October 6th at hand. In regard to "Are amalgam fillings injurious?" I can say that for the past seven years, since my attention was called to the subject by Prof. Eugene W. Sawyer and the late Dr. E. A. Ballard, my early prejudices were soon removed, and for five or six years I have become more fully and constantly convinced that amalgams and red vulcanite are seriously affecting the mental and physical health of a large majority of the people who come to the doctor for his medical and often surgical aid. A large class of neuralgias, dental, facial and otherwise—as intercostal, ovarian, pluritic or gastric have been very materially lessened if not cured by removal of the amalgams—especially—and more surely *cured* when black vulcanite plates have been substituted for the red.

Amalgam especially favors, and in many instances *causes*, headaches, faceaches, nervousness and melancholia, repeated and chronic attacks of tonsillitis, aphthæ, and ulceration of edges of tongue, sore and bleeding gums and rheumatoid pains *anywhere*—and I have seen hæmorrhoids, which ordinary treatment failed to cure, promptly and permanently disappear—after removing the amalgams. I have tested these various cases to my satisfaction by treating the patient with placebo while observing the behavior of the cases under simple removal of the amalgam. One case, in particular, I will condense. G—. S—., aged twenty-four, blonde, vocalist, for many months had to give up her art because of repeated attacks of hoarseness, toothache often accompanying, and a constantly offensive breath, disturbance of menstrual functions, irregular, scanty, painful—later laryngitis, with enlarged tonsils in periodical attacks, worse during the menstrual visitation, a constantly increasing melancholia as she became discouraged from repeated failure of different doctors to cure. Her awful dread of operation had saved to her the tonsils. Through several months I treated her case with indifferent success—and from my viewpoint pronounced failure—only palliating. I finally broke through my thin prejudice, and insisted upon removal of the several large amalgams, (all antedating the vocal and other troubles) substituting gold or cement. This was thoroughly done by the dentist, though he volunteered the information that her doctor was a crank of the first-water. The results were prompt. Improvement went steadily on until she was restored to fairly good health. Her voice returned—functions regained their physiological regularity, and she was and is still a bright and happy woman. This case for several weeks following "de-amalgamating" process I treated with saccharum lactis and also it removed a large amount of my prejudice. I am glad to give my mite to this subject. Dr. Sawyer, myself and an increasing many are doing our best to hammer this philosophy into the profession, but alas, that element of "hide bound" prejudice against the doctrine of infinitesimals is a great stumbling stone in the way of progress. If they would only put the matter to the "test of experience" and observation. I could go on and give you more cases not only of the amalgam evil but that of the red vulcanite, but this must suffice for this time.

Sincerely,

F. O. Pease.

---

Samuel A. Kimball, M. D.,
124 Commonwealth Ave.,
Boston, Mass., January 23 1898.

Charles H. Taft, D. M. D.

Dear Doctor: In reply to your request for information in regard to Mr. R— P—, I would say that he has been under my observation and treatment for years for catarrhal affections of the throat and nose. There were enlarged tonsils and probably adenoid growths in the pharynx, as when the tonsils at times were not much enlarged there would still be mouth breathing and indications of nasal obstruction. He took cold easily and then his tonsils would swell so as to meet. There would be a profuse discharge from the nose and throat, and after awhile improvement would begin and he would be relieved until he contracted another cold. He was never free from more or less swelling of the tonsils, obstruction of the nose, post nasal discharge of mucous and breathing through his mouth most of the time, always during sleep. In other respects he was very well. I told his parents that he would not recover from this condition of affairs until his amalgam fillings were removed; that if this were not done only temporary relief would be obtained. After several years' delay, for of course they didn't believe it, they at last consented as the boy's condition was becoming worse. You removed them, I believe, in April, 1896. I have seen the boy but once since; then when he came in on April 25, 1896, to tell me that you had taken out the fillings. He has not required my services since, having had no colds, so his mother reports, no swellings of the tonsils, no catarrh, and a perfectly healthy young man in ever way.

Sincerely yours,

S. A. Kimball.

---

### Letters from Patients.

Boston, October 4, 1897.

My Dear Dr. Taft: Your note was received a day or two ago and I will do my best to give you a satisfactory account of myself "before and after" the amalgam fillings were removed. I had, according to accounts, something over twenty amalgam fillings in my teeth. For some years the mucous membrane of my throat was affected to such an extent that it was difficult for me to swallow. The lumps that formed made a most offensive taste in my mouth and odor to my breath. These lumps came and went constantly. After an attack of the grippe, Dr. Allen told me he could do much more towards a rapid recovery and a strong constitution if I would but have the amalgams removed, which I did as soon as I was able to sit up three hours at a time. After the deed was done Dr. Allen's medicine seemed to have immediate effect, and I was soon entirely well. My throat has given me trouble but four times since then, as far as I can remember—and then *very* slight attacks, so to speak, and the trouble in the throat readily traced to some cold. As this change was made in the spring of '95 and "no trouble" has taken the place of "constant trouble" in that time, I think, undoubtedly, they were of great harm; and since then have tried to induce others to have their amalgams removed, because of my own personal convictions in that line. Dr. Allen has told me since then that it has been much easier for his medicines to act—and thinks added strength was due to that. Of course you understand, I am only too glad to have these evidences used if they can be any good to another, but would prefer not to have any name attached.

Hoping this is satifactory, I am,

Very sincerely yours,

———

NEWTON, MASS., October 20, 1897.

DR. TAFT.

DEAR SIR: You may perhaps be glad to learn if I can see any good results from the removal of my amalgam fillings. For the past eight or ten years I have had very severe nervous headaches and a great deal of insomnia. At first medicine would relieve, but later seemed to have no effect whatever. Dr. McIntosh thought, from certain symptoms, that I had mercurial poison in my system and ordered amalgam fillings removed. I had reached the point where I was willing to try almost anything, although candidly, when I went to you in the winter I hardly dared hope for improvement. For the first three or four months I could see no change: Since then I have been slowly and *surely* gaining. I sleep well, have very few headaches and when obliged to resort to medicine am quickly affected by it. My general health is better in every way. I asked Dr. McIntosh yesterday if he thought all this the result of having the fillings removed and he did not hesitate to say "Yes." Certainly *I* am convinced and am more than pleased to relate my experience to anyone who is interested. I heard the subject discussed while away this summer, and find that there are many who now consider amalgam injurious. I feel grateful to Dr. McIntosh for advising, and to you for carrying out the advice. I don't suppose the effects are entirely gone or will be for some time, but even now I am repaid.

Yours very sincerely,

———.

CHICAGO, October 5, 1897.

DEAR DOCTOR: In reply to your letter of the 1st inst. in which you ask me to write you of the condition of my health since the removal of the amalgam fillings, will say that I have steadily and greatly improved in health under treatment, but how much credit is due to the gold fillings or to the removal of the amalgams I am at a loss to tell as I had been under the doctor's care sometime before you removed the amalgams. However, while the amalgams were in my teeth I had a decided and ever present metallic taste in my mouth, and on arising in the morning my mouth seemed filled with this taste which no amount of cleaning with brush and powder would wholly take away. This, however, disappeared with the amalgams. My tongue also showed the imprints of the teeth and does some yet but are going away slowly. The original cause for which I consulted the doctor is for tumor on the neck, which tumor is still in my possession, but gives me no inconvenience. I was also suffering from severe and continued headaches, bleeding piles, constipation and some other troubles which I do not now recall. My headaches are now a thing of the past. Very seldom any trouble from piles, and the trouble with my bowels has been corrected and are now regular, and I am now feeling fine with the exception of a little rheumatism in my ankels and feet but hope soon to be rid of this also. The baneful influence of the amalgam fillings permeated my entire system and aggravated my other troubles. I wish I could give you a clearer case or one in which results were more apparent.

Yours very truly,

———.

---

BOSTON, October 5, 1897.

DEAR DOCTOR: I am in receipt of your favor of the 1st inst. and in reply will say my general health is better since the amalgam fillings were removed. My weight has increased some eight or ten pounds, which is remarkably good for me, for I do not think it had varied two pounds in the five or six years previous to having them removed. I hardly know how to go into particulars regarding my health as I was never subject to headaches or anything of that kind, and the improvement has been in my general condition. I am of the opinion that my blood is in better condition, and I am very certain that I can do more work and not feel so tired as I did have a couple of years ago. Assuring you that I shall be very glad to answer any questions that may occur to you,

I remain, very sincerely yours,

———————.

SOMERVILLE, MASS., October 4, 1897.

DEAR DR. TAFT: I received your letter Saturday, and I am very glad to give you the report for which you ask. When Dr. Thurston first began treating me, four years ago, I suffered much of the time from canker in my mouth. I also had an offensive breath most of the time and a disagreeable taste in the mouth. Dr. Thurston insisted that all these things were caused by the amalgam fillings in my teeth and urged me to have them taken out, but I was skeptical and did nothing about the matter for two years. During that time I did not improve very much under Dr. Thurston's treatment, and at last decided to have the fillings taken out. Since they were removed I have had *very* little canker, my breath has not been offensive at all, no excessive amount of saliva as before, and the disagreeable taste in my mouth has entirely disappeared. I firmly believe *now* that the amalgam fillings were the cause of all the trouble, and I shall never have any more of them put into my teeth. My general health is considerably improved since I saw you last, but I am still far from strong.

Very truly yours,

———————.

BOSTON, MASS., October 19, 1897.

CHARLES H. TAFT, D.M.D., 16 Arlington St., Boston, Mass.

DEAR DOCTOR: I have just returned from the West, and among other papers on my desk found your letter asking my experience and feeling towards amalgam filling. I will say in answer, that I am disgusted with the stuff, and have no use (as a dentist) for a dentist who advocates and advises its use. It had caused me great suffering, and I am thankful that not a particle of it is in my teeth to-day. It seems to me that, with so much progress in other things, dentists should have discovered long before this a substitute for gold, which should be lasting and preserving to the teeth, which would come within the reach of the thousands who cannot afford gold, but who should have their teeth when once filled, filled for all time.

